# Early crestal bone loss: Is it really loss?

**DOI:** 10.1002/ccr3.2376

**Published:** 2019-08-27

**Authors:** Algirdas Puisys, Viktorija Auzbikaviciute, Agne Minkauskaite, Renata Simkunaite‐Rizgeliene, Dainius Razukevicius, Rokas Linkevicius, Tomas Linkevicius

**Affiliations:** ^1^ Vilnius Implantology Center Vilnius Lithuania; ^2^ Vilnius Research Group Vilnius Lithuania; ^3^ Vilnius University Vilnius Lithuania; ^4^ Lithuanian University of Health Sciences Kaunas Lithuania

**Keywords:** bone remineralization, bone stability, crestal bone loss

## Abstract

Bone remineralization around dental implants might be possible after early crestal bone loss.

## 1 INTRODUCTION

Early crestal bone loss is defined as a bone resorption around dental implant neck within 1 year postloading. Due to its frequency, the certain amount of bone loss is becoming a norm. Albrektsoon et al in 1896,^1^ already stated that 1.5 mm of bone loss at the 1 year after loading can be considered as a success, if later bone loss does not exceed 0.2 mm annually. Even though this statement was three decades ago, the clinicians still keep it as a normal bone remodeling process.

However, not stable bone may cause different problems, leaving the clinician uncertain, if the implant will be stable for longer. For this reason, clinician's duty is to seek as least bone loss as possible.

Within the 1 year of loading, bone matures and becomes more dense, and occlusal forces that initially cause crestal bone loss are not great enough to evoke further bone resorption. The question arises, is it possible not only to prevent bone resorbtion but also anticipate bone remineralization with increased crestal bone around dental implant?

This article describes several clinical cases where we observed bone remineralization around implants within two years history of early crestal bone loss.

## 2 CLINICAL CASES

In this article, we are going to present you 3 different clinical cases of early crestal bone loss and bone remineralization.

### 2.1 Case 1

**Figure 1:**
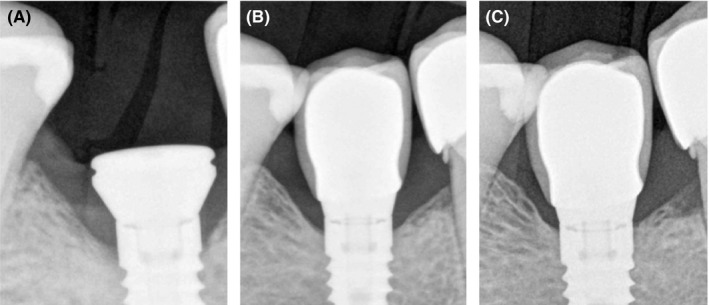
A, Early crestal bone loss after implant placement at 2011. 89 × 119 mm (72 × 72 DPI). B, Implant loading at 2012. 88 × 119 mm (72 × 72 DPI). C, Bone remineralization after 2 y (2014). 89 × 119 mm (72 × 72 DPI)

Bone level (Biohorizons) implant was placed at the site of #46 tooth simultaneously with healing abutment. After 3 months of osseointegration (Figure 1A), metal ceramic crown was placed with a regular platform (Figure 1B). During regular checkup, bone remineralization around dental implant #46 is visible together with stable crestal bone (Figure 1C).

### 2.2 Case 2

**Figure 2:**
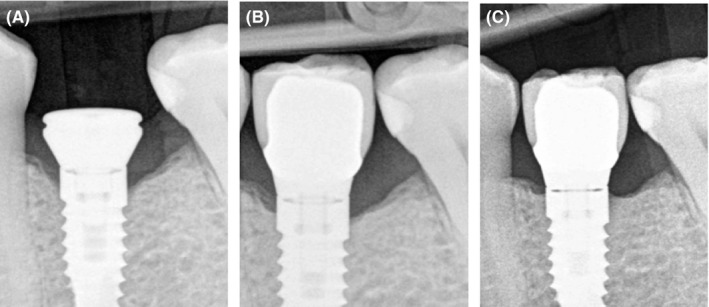
A, Early crestal bone loss after implant placement at 2011. 88 × 119 mm (72 × 72 DPI). B, Implant loading at 2012. 88 × 118 mm (72 × 72 DPI). C, Bone remineralization after 2 y (2014). 88 × 118 mm (72 × 72 DPI)

Another clinical case shows similar postop result. Bone level (Biohorizons) dental implant was placed nonsubmerged. Periapical X‐ray before prosthetic treatment shows early crestal bone loss (Figure 2A). After implant loading with a metal ceramic crown and regular platform, bone remains similar with previous observations (Figure 2B). Two years later, periapical radiograph was made and it shows bone remineralization at the site #36 (Figure 2C). Crestal bone contour is stable.

### 2.3 Case 3

**Figure 3:**
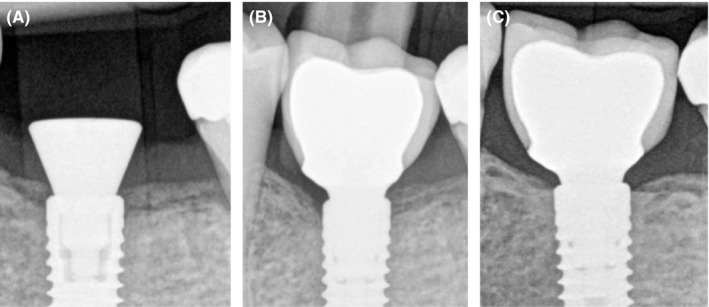
A, After implant placement at 2012. 89 × 118 mm (72 × 72 DPI). B, Implant loading three months later. 87 × 118 mm (72 × 72 DPI). C, Bone remineralization after 2 y (2014). 88 × 118 mm (72 × 72 DPI)

Third clinical case: Patient came to the clinic for the implant placement at the site #36. Bone level (Straumann) dental implant was placed, and healing abutment was screwed at the same surgery. Three months later, periapical X‐ray reveals pleasant results but still early crestal bone loss is visible (Figure 3A). During prosthetic treatment, implant was loaded with metal ceramic crown and platform switching (Figure 3B). Two years later, a regular checkup and new periapical X‐ray were made. It is seen that bone remineralization occurred also in this case with a stable crestal bone around implant neck (Figure 3C).

## 3 DISCUSSION

Three similar cases were showed with visible crestal bone loss and following remineralization process around dental implants. With this in mind, it can be presumed that not always crestal bone loss is real bone resorption of the bone tissue. In some cases, only demineralization of crestal bone happens, looking like a bone loss in periapical X‐ray.

Demineralization is the process caused by inflammation when mineral ions of hydroxyapatite (HA) removed from the hard tissues, particularly in the bone.^2^ Even though HA is one of the most stable calcium phosphate salt,^3^ the inflammation process might lead to the bone matrix changes following bone loss. Inflammation is related with the overproduction of various cytokines and bone cells.^4^ They initiate hyperactivation of osteoclasts and lead to the bone degradation; also, some cytokines negatively affect osteoblast function.^5^ Remineralization might be achieved by increasing osteoblasts function; particularly, these cells promote crystal formation of hydroxyapatite, propagate growth in the interior part of membrane‐limited matrix vesicles,^6^ and induce crystals in the collagenous extracellular matrix thus mineralizing bone matrix overall.^7^

There are many factors influencing early crestal bone loss. The most important are platform switching, polished implant neck, stable connection, and sufficient vertical soft tissue thickness around implant neck.^8^ Paying attention to these factors, it seems possible to have a huge potential for zero bone loss or even bone remineralization, if it happens. Previous option is already seen in our clinical practice.

As a weakness of this statement could be mentioned accuracy of periapical X‐rays, the state of being parallel and equally angulated.

Bone remineralization may occur around dental implants with a history of early crestal bone loss after loading. There is still considerable uncertainty of exact histological process of bone matrix around dental implant neck. In order to have comprehensive evidence‐based analysis, further experimental investigations are needed to estimate accurate results.

## CONFLICT OF INTEREST

None declared.

## AUTHOR CONTRIBUTION

PA: was responsible for the surgery part of the treatment; AV: interpreted the patient data, designed, and wrote the manuscript; MA: was in charge for the data collection; SRR: involved in description of histological part for the remineralization process; RD: analyzed data; LR: designed the manuscript; TL: performed prosthetic part of the treatment.
